# Stroke Care During the First and the Second Waves of the COVID-19 Pandemic in a Community Hospital

**DOI:** 10.3389/fneur.2021.655434

**Published:** 2021-08-02

**Authors:** Piotr Sobolewski, Wiktor Szczuchniak, Danuta Grzesiak-Witek, Jacek Wilczyński, Karol Paciura, Mateusz Antecki, Tadeusz Frańczak-Prochowski, Marek Kos, Grzegorz Kozera

**Affiliations:** ^1^Department of Neurology and Stroke Unit in Sandomierz, Jan Kochanowski University, Kielce, Poland; ^2^Collegium Medicum, Jan Kochanowski University, Kielce, Poland; ^3^Institute of Literature and Linguistics, Jan Kochanowski University, Kielce, Poland; ^4^Laboratory Diagnostics, Holy Spirit Specialist Hospital in Sandomierz, Sandomierz, Poland; ^5^Department of Public Health, Medical University of Lublin, Lublin, Poland; ^6^Medical Simulation Center, Medical University of Gdańsk, Gdańsk, Poland

**Keywords:** COVID-19, two waves of pandemic, cerebrovasacular events, community hospital, personnel infections

## Abstract

**Objective:** The coronavirus disease 2019 (COVID-19) infection may alter a stroke course; thus, we compared stroke course during subsequent pandemic waves in a stroke unit (SU) from a hospital located in a rural area.

**Methods:** A retrospective study included all patients consecutively admitted to the SU between March 15 and May 31, 2020 (“first wave”), and between September 15 and November 30, 2020 (“second wave”). We compared demographic and clinical data, treatments, and outcomes of patients between the first and the second waves of the pandemic and between subjects with and without COVID-19.

**Results:** During the “first wave,” 1.4% of 71 patients were hospitalized due to stroke/TIA, and 41.8% of 91 during the “second wave” were infected with SARS-CoV-2 (*p* < 0.001). During the “second wave,” more SU staff members were infected with COVID-19 than during the “first wave” (45.6 vs. 8.7%, *p* < 0.001). Nevertheless, more patients underwent intravenous thrombolysis (26.4 vs. 9.9%, *p* < 0.008) and endovascular thrombectomy (5.3 vs. 0.0%, *p* < 0.001) during the second than the first wave. Large vessel occlusion (LVO) (OR 8.74; 95% CI 1.60–47.82; *p* = 0.012) and higher 30-day mortality (OR 6.01; 95% CI 1.04–34.78; *p* = 0.045) were associated with patients infected with COVID-19. No differences regarding proportions between ischemic and hemorrhagic strokes and TIAs between both waves or subgroups with and without COVID-19 existed.

**Conclusion:** Despite the greater COVID-19 infection rate among both SU patients and staff during the “second wave” of the pandemic, a higher percentage of reperfusion procedures has been performed then. COVID-19 infection was associated with a higher rate of the LVO and 30-day mortality.

## Introduction

The coronavirus disease 2019 (COVID-19) pandemic is associated with altered course of cerebrovascular diseases. COVID-19-associated hypercoagulability that is likely a “sepsis-induced coagulopathy” also predisposes to stroke (1). The heterogeneity of stroke etiology and clinical picture in the course of COVID-19 infection is emphasized. The complex mechanisms of COVID-19-induced vascular injury, resulting from non-specific effects of inflammation and endothelial dysfunction and coagulation disorders, also impose on pre-existing cerebrovascular risk factors (1, 2).

The COVID-19 pandemic started in China, and consequently, the physicians from Wuhan first reported strokes in infected patients (3). Later publications confirmed these reports (4, 5). The rates of stroke in hospitalized patients with COVID-19 from 1 to 3% and up to 6% of critically ill patients were reported (3, 6–8). Cerebrovascular events in patients with COVID-19 are mainly ischemic, but hemorrhagic strokes and cerebral venous sinus thrombosis, especially in critically ill patients, have also been described (9).

The COVID-19 pandemic has varied in severity since the beginning of 2020 in each country or region. The pandemic affecting all parts of the world is also having huge implications for stroke care. Based on the results of the World Stroke Organization survey, it is known that during the COVID-19 pandemic, there was a decrease in hospitalizations due to acute stroke, especially of patients with milder stroke. This trend did not depend on differences in national health systems (10). The pandemic may also influence the way of care delivery in acute stroke. During the pandemic, it becomes more difficult to meet the requirements of the current guidelines of scientific societies regarding the diagnosis and treatment of acute stroke patients.

Thus, the paper presents two significantly different faces of the COVID-19 pandemic waves, in the months from March to May and from September to November 2020, in relation to the diagnosis and treatment of patients with acute stroke in a hospital located in the rural region of south-east Poland.

## Materials and Methods

### Study Design

We conducted a retrospective observational study based on a hospital-based stroke registry from the Department of Neurology and Stroke Unit of Holy Spirit Specialist Hospital in Sandomierz. Our center is recognized as a stroke unit according to Polish national criteria, equipped with the proper monitoring and diagnostic facilities and provides a 24-h stroke service 7 days a week (11). In the stroke unit, diagnostic and treatment procedure protocols with respect to unified regular protocols of the management of acute IS and secondary prevention, according to international recommendations, are in force (12, 13).

### Study Population

All consecutive acute stroke and transient ischemic attack (TIA) patients admitted to the stroke unit in Sandomierz between March 15, 2020, and May 31, 2020 and between September 15, 2020, and November 30, 2020 were included. In terms of treating strokes, the hospital covers a population of approximately 160,000 inhabitants from the Sandomierz, Opatów, and Tarnobrzeg regions.

A stroke physician examined all patients at the time of admission, and the severity of stroke symptoms was assessed using the National Institute of Health Stroke Scale (NIHSS) (14). Stroke onset was defined as the last occasion on which the patient was known to be without neurological deficit. Exams to evaluate the inflammatory processes and the coagulation status in all patients and also chest X-ray and/or chest computed tomography (CT) were performed. Brain CT and/or magnetic resonance (MR) was performed upon admission to the hospital in order to establish the indication for treatment and between 22 and 36 h and on the seventh day in patients who underwent i.v. thrombolysis. To evaluate the etiology of the stroke, transcranial Doppler (TCD), carotid duplex ultrasonography, Holter electrocardiography (Holter ECG), and transthoracic echocardiography (TTE) were performed. Stroke diagnosis has been established based on ICD-10 criteria.

The 30-day stroke outcomes were measured using the modified Rankin scale (mRS) (15). A favorable outcome was defined as an mRS score ≤ 2 points, while an unfavorable outcome was defined as an mRS score of 3–6 points.

The ethics committee approved all data analysis (Ethics Committee of Jan Kochanowski University in Kielce).

### Statistical Analysis

This study was based on a retrospective data analysis. Data collection, descriptive statistics, and univariate analysis were performed using Microsoft Excel 2019. In cerebrovascular patients, comparisons in terms of demographics, effectiveness, and safety of treatment between subgroups—patients treated during the first and the second waves of the COVID-19 pandemic and those with and without COVID-19 infection—were performed. Logistic regression was performed with STATISTICA v. 9.1. All continuous variables were tested for a normal distribution and equality of variances. Because of the non-normality of the variables, non-parametric Mann–Whitney *U* tests were used to perform the univariate analysis of the continuous variables. Categorical data were compared using chi-square tests; *p* < 0.05 were considered statistically significant. The multivariate analysis was performed using multiple logistic regression models. Factors identified in the univariate analysis with a *p* < 0.05 were then examined using a multivariate model.

## Results

There were more cerebrovascular incidents treated in the stroke unit during the second wave of the pandemic than during the first wave (91 vs. 72, *p* = 0.034). The univariate analysis showed that both groups did not differ in terms of demographic data, risk factors, type of cerebrovascular event, symptoms of stroke or TIA, 30-day efficacy of treatment, and mortality (Table 1).

**Table 1:** The clinical characteristics of the subgroups of patients treated during the first and second wave of the COVID-19 pandemic.

**Variables**	**The first wave of pandemic**	**The second wave of pandemic**	***P* Value**
**Number of days of pandemic**	**78**	**77**	
*n* (%)	71 (43.83)	91 (56.17)	<0.001
**Demographic data**			
Age (years) mean, SD	75.70 (12.43)	73,78 (12.81)	0.338
Male gender, *n* (%)	39 (53.94)	40 (43.96)	0.165
**Risk factors**, ***n*****(%)**			
Baseline mRS 3–5	7 (9.86)	11 (12.09)	0.654
Arterial hypertension	54 (76.06)	63 (69.23)	0.336
Atrial fibrillation	20 (28.17)	19 (20.88)	0.281
Diabetes mellitus	13 (18.31)	14 (15.38)	0.620
**Type of cerebrovascular event, n (%)**			
Ischemic stroke	50 (70.42)	75 (82.42)	0.071
Intracerebral hemorrhage	7 (9.86)	6 (6.59)	0.448
TIA	14 (19.72)	10 (10.99)	0.121
**Symptoms of stroke or TIA, n (%)**			
from the anterior circulation	51 (71.83)	72 (79.12)	0.281
from the posterior circulation	13 (18.31)	8 (8.89)	0.078
disturbances of consciousness	11 (15.49)	7 (7.69)	0.117
**Etiological classification, n(%)**			
Large vessel occlusion	0	31 (54.67)	***<0.001***
Cardioembolism	16 (32.00)	11 (13.75)	**0.013**
Lacunar stroke	14 (28.00)	18 (22.50)	0.479
Undetermined etiology	4 (8.00)	5 (6.25)	0.702
**COVID-19 infection, n (%)**			
Symptomatic COVID with lung infection	1 (1.41)	19 (20.88)	***<0.001***
Confirmed COVID-19 infection	1 (1.41)	38 (41.76)	***<0.001***
Before cerebrovascular event	1 (141)	33 (36.26)	***<0.001***
During cerebrovascular event	0	8 (8.79)	0.010
**NIHSS on admission, [**points] **median (IQR)**	6.00 (2.00–12.00)	6.00 (2.00–12.00)	0.783
**Laboratory findings, median (IQR)**			
Hemoglobin level [g/dL]	13.85 (13.10–15.10)	13.50 (12.70–14.60)	***0.049***
White blood cells [x10^9^/L]	8.30 (6.90–11.90)	8.60 (7.00–10.40)	0.444
Platelets [x10^9^/L]	225.00 (187.00–280.00)	219.00 (172.00–270.00)	0.300
Fibrinogen level [mg/dL]	357.00 (304.00–433.00)	391.00 (332.00–495.00)	***0.018***
D–dimer level [ng/mL]	878.00 (400.00–2697.00)	724.00 (528.00–1656.00)	0.967
C–reactive protein level [mg/L]	9.00 (2.80–78.80)	14.15 (2.60–56.50)	0.869
Procalcitonin level [ng/mL]	0.16 (0.04–0.22)	0.05 (0.02–0.12)	***0.020***
**Secondary prophylactic**, ***n*****(%)**			
VKA	1 (1.41)	3 (3.30)	0.442
NOAC	11 (15.49)	6 (6.59)	0.067
Antiplatelets	46 (64.79)	41 (45.05)	***0.012***
LMWH	4 (5.63)	6 (6.59)	0.801
Antiplatelets + LMWH	6 (8.45)	28 (30.77)	***<0.001***
**Intracerebral thrombolysis**, ***n*****(%)**	7 (9.86)	24 (26.37)	**0.008**
Onset-to-treatment time [min.]	143.00 (123.00–165.00)	137.50 (105.50–205.00)	0.962
Door-to-treatment time [min.]	54.00 (45.00–66.00)	45.00 (30.00–89.50)	0.636
PH2, *n* (%)	1 (14.29)	1 (4.17)	0.338
SICH^‡^, *n* (%)	1 (14.29)	0	0.060
**Mechanical thrombectomy**, ***n*****(%)**	0	4 (5.33)	***<0.001***
Onset-to-ship time [min.]	-	175.00 (127.00–230.00)	–
**mRS 0–2 at 30-day**, ***n*****(%)**	31 (43.66)	47 (51.65)	0.313
**30-day mortality**, ***n*****(%)**	11 (15.49)	17 (18.68)	0.549

‡ *According to the ECASS II and III criteria; TIA, Transient Ischemic Attack; mRS, modified Rankin scal; NIHSS, National Institutes of Health Stroke Scale ECASS, European Cooperative Acute Stroke Study; SICH, Symptomatic Intracerebral Hemorrhage; VKA, vitamin K antagonists; NOAC, novel oral anticoagulants; LMWH, Low-molecular-weight heparin; SD, standard deviation; IQR, interquartile range(Q_1_-Q_3_). The bold valued show the significance of the p < 0.05*.

During the first wave of the COVID-19 pandemic, only 1.4% of patients were infected compared with 41.8% during the second wave (*p* < 0.001). Patients treated during the second wave of the pandemic were characterized by more frequent large vessel occlusion (LVO) (*p* < 0.001), higher level of fibrinogen (*p* = 0.018), less frequent diagnosis of cardioembolic stroke (*p* = 0.013), and lower level of hemoglobin (*p* = 0.048) and procalcitonin (*p* = 0.02) compared with patients from the first wave. During the second wave of the pandemic, more patients underwent intravenous thrombolysis (IVT) (26.4 vs. 9.9%, *p* < 0.008) and endovascular thrombectomy (EVT) (5.3 vs. 0.0%, *p* < 0.001). There was no difference in terms of the logistic times: onset-to-treatment time (OTT) (*p* = 0.96) and door-to-needle time (DNT) (*p* = 0.64) (Table 1). During both waves of the COVID-19 pandemic, regular physician care for stroke patients was reported to be only 23.1% compared with previous data from our stroke register–59.9% (*p* < 0.001).

During the second wave of the pandemic, more of the stroke unit personnel were infected with COVID-19 than during the first wave (42.6 vs. 8.7%, *p* < 0.001).

The univariate analysis showed that the group of cerebrovascular patients infected with COVID-19 was characterized by more frequent diagnosis of LVO (*p* < 0.001), higher C-reactive protein (CRP) level (*p* = 0.002), higher 30-day mortality (*p* = 0.03), and lower hemoglobin level (HGB) (*p* = 0.015), white blood cells (WBC) (*p* = 0.014), and platelet counts (*p* = 0.04). There was no difference in the percentage of treatment with IVT (*p* = 0.42) and in terms of OTT (*p* = 0.86) and DNT (*p* = 0.52) in both groups; however, in patients with COVID-19, EVT was more frequent (*p* = 0.009) (Table 2).

**Table 2:** The clinical characteristics of the subgroups of patients with cerebrovascular diseases and COVID-19 infection.

**Variables**	**The patients with COVID-19 infection**	**The patients without COVID-19 infection**	***P* Value**
*n* (%)	38 (23.46)	124 (76.54)	—
**Demographic data**			
Age [years] mean, SD	75.05 (13.46)	74.49 (12.44)	0.812
Male gender, *n* (%)	16 (42.11)	63 (50.81)	0.348
**Risk factors**, ***n*****(%)**			
Baseline mRS 3–5	5 (13.16)	13 (10.48)	0.646
Arterial hypertension	27 (71.05)	90 (72.58)	0.854
Atrial fibrillation	8 (21.05)	31 (25.00)	0.618
Diabetes mellitus	6 (15.79)	21 (16.94)	0.868
**Type of cerebrovascular event**, ***n*****(%)**			
Ischemic stroke	32 (84.21)	93 (75.00)	0.237
Intracerebral hemorrhage	1 (2.63)	12 (9.68)	0.162
TIA	5 (13.16)	19 (15.32)	0.724
**Symptoms of stroke or TIA**, ***n*****(%)**			
From the anterior circulation	30 (78.95)	93 (75.00)	0.618
From the posterior circulation	2 (5.26)	19 (15.45)	0.103
Disturbances of consciousness	4 (10.53)	14 (11.29)	0.896
**Etiological classification**, ***n*****(%)**			
Large vessel occlusion	19 (58.38)	22 (23.66)	***<0.001***
Cardioembolism	5 (15.15)	22 (22.68)	0.357
Lacunar stroke	4 (12.12)	28 (28.87)	0.053
Undetermined etiology	4 (12.12)	5 (5.15)	0.173
**NIHSS on admission, [**points] **median (IQR)**	9.50 (3.00–15.00)	5.00 (2.00–12.00)	0.110
**Laboratory findings, median (IQR)**			
Hemoglobin level [g/dL]	13.30 (12.20–14.00)	13.90 (12.80–15.00)	***0.015***
White blood cells [x10^9^/L]	7.75 (6.40–9.10)	8.70 (7.25–11.60)	***0.014***
Platelets [x10^9^/L]	194.00 (149.00–270.00)	229.00 (190.50–278.50)	***0.041***
Fibrinogen level [mg/dL]	411.00 (332.00–495.00)	367.00 (315.00–458.00)	0.086
D-dimer level [ng/mL]	1134.00 (668.00–1656.00)	698.00 (400.00–1819.00)	0,057
C-reactive protein level [mg/L]	47.70 (7.80–72.00)	7.25 (2.20–43.40)	***0.002***
Procalcitonin level [ng/mL]	0.11 (0.04–0.18)	0.04 (0.02–0.16)	0.070
**Secondary prophylactic**, ***n*****(%)**			
VKA	0	4 (3.23)	0.262
NOAC	1 (2.63)	16 (12.90)	0.071
Antiplatelets	13 (34.21)	74 (59.68)	***0.006***
LMWH	4 (10.53)	6 (4.84)	0.202
Antiplatelets + LMWH	17 (44.74)	17 (13.71)	***<0.001***
**Intracerebral thrombolysis**, ***n*****(%)**	9 (23.68)	22 (17.74)	0.415
Onset-to-treatment time [min.]	135.00 (115.00–190.00)	141.50 (120.00–175.00)	0.861
Door-to-treatment time [min.]	40.00 (23.00–59.00)	49.00 (34.00–72.00)	0.514
PH_2_, *n* (%)	0	2 (9.09)	0.350
SICH^‡^, *n* (%)	0	1 (4.55)	0.516
**Mechanical thrombectomy**, ***n*****(%)**	2 (20.00)	2 (2.35)	***0.009***
Onset-to-ship time [min.]	230.00 (210.00–250.00)	127.00 (114.00–140.00)	0.121
**mRS 0–2 at thirty-day**, ***n*****(%)**	15 (39.47)	63 (50.81)	0.221
**30-day mortality**, ***n*****(%)**	11 (28.95)	17 (13.71)	***0.030***

‡ *According to the ECASS II and III criteria; TIA, Transient Ischemic Attack; mRS, modified Rankin scal; NIHSS, National Institutes of Health Stroke Scale ECASS, European Cooperative Acute Stroke Study; SICH, Symptomatic Intracerebral Hemorrhage; VKA, vitamin K antagonists; NOAC, novel oral anticoagulants; LMWH, Low-molecular-weight heparin; SD, standard deviation; IQR, interquartile range(Q_1_-Q_3_). The bold valued show the significance of the p < 0.05*.

The multivariate analysis showed that diagnosis of LVO (OR 8.74; 95% CI 1.60–47.82; *p* = 0.012) and higher 30-day mortality (OR 6.01; 95% CI 1.04–34.78; *p* = 0.045) were associated with patients infected by COVID-19 (Table 3).

**Table 3:** Multivariate logistic regression models showing confounders of clinical characteristics of stroke with COVID-19 infection.

**Variables**	**COVID-19 infection**		
	**OR**	**95% CI**	***p*-value**
Hemoglobin level	0,84	0,48–,46	0,534
Platelets	1,01	0,99–1,02	0,267
Large vessel occlusion	***8,74***	***1,60–47,82***	***0,012***
Mechanical thrombectomy	6,15	0,45–84,20	0,173
30-day mortality	***6,01***	***1,04–34,78***	***0,045***

*The bold valued show the significance of the p < 0.05*.

## Discussion

We showed that the courses of the two waves of the pandemic were different in terms of the workload of the stroke unit of a rural hospital located in south-eastern Poland. In Poland, the number of COVID-19 infections per million inhabitants during the corresponding periods of the two waves of the pandemic was 20 and more than 25,500, respectively (16, 17). In the adequate periods of 2019, 88 and 93 patients with cerebrovascular incidents have been hospitalized in our stroke center (the IVT rate and DNT were 29.3%/40 min and 36%/37 min, respectively). EVT was not available in our center in 2019 due to the fact that the interventional center for our region was being organized then. During the first wave of the pandemic, we hospitalized about one-fifth stroke patients less, the percentage of IVT was three times lower, and DNT was longer than in previous years in a similar period. However, analogous data from the second wave of the pandemic were comparable to the data from the 2019 period.

During the first wave of the pandemic, although the number of patients infected with SARS-CoV-2 was small and although the activities of the neurology department were disturbed, there were interruptions in admitting patients to the ward, and the medical staff were repeatedly quarantined. This is consistent with previous reports of observational studies that hospitals significantly reduced care delivery in response to the first wave of the COVID-19 pandemic (18–20). Similar observations apply to the care of patients with stroke (10). Certainly, at the time of the first wave of the pandemic, the number of cases with confirmed COVID-19 infection was underestimated. This was due to the lack of experience of the medical personnel in diagnosing the disease as well as the unavailability and imperfection of diagnostic tests (21–23).

As in other European countries, in September and October in Poland, there was a sharp increase in morbidity and deaths due to COVID-19 (22, 24). At the same time, there was a significant increase in the incidence of cerebrovascular events (24).

In our group of patients with COVID-19, in some of them, stroke occurred on consecutive days of infection, while the remaining patients developed infection in the first few days of stroke. Previous studies reported that the median incidence of stroke from onset of infection is 10 days (25). Patients with acute stroke may be asymptomatic carriers in the prodromal period, and neurological deficits are the first manifestation of COVID-19 infection (26).

The results of several studies showed that a significant group among the infected was patients with LVO, which could be explained by the coagulation and microcirculation disorders described in patients with COVID-19 (27, 28). In our group of ischemic stroke patients with COVID-19, patients with diagnosis of LVO accounted for nearly 60% of the entire infected group. However, during the first wave of the pandemic, the lower number of strokes and the lack of patients with major strokes due to LVO could be explained by the limited transport from other hospitals to our stroke center. The necessity of its improvement was one of the main lessons learned both from the first and the second waves of the pandemic. It should also be noted that the proportion of cryptogenic strokes was not different between infected and non-infected patients, which contradicts the results of other studies (29, 30).

Despite the greater number of COVID-19 infections during the second wave of the pandemic, the organization of work at the emergency and stroke units was improved, which resulted in a high percentage of reperfusion therapy in patients with ischemic stroke. We do not have information on the use of reperfusion therapies during the second wave of the pandemic; however, the published data on the use of IVT and EVT during the first wave indicated significant limitations in the use of these therapies (5, 18–20, 25).

In the field of laboratory tests, patients with COVID-19 were characterized by a higher level of fibrinogen and CRP and a lower level of HGB, platelet, and WBC counts. We did not find a difference in the level of D-dimer, but the results significantly exceeded the laboratory norm in both groups. Our findings are consistent with the results of previous observational studies, in which a high level of fibrinogen and D-dimer and a low level of platelets in patients with cerebrovascular diseases infected by SARS-CoV-2 were pointed out (5, 31–33).

In the group of patients with cerebrovascular diseases infected with COVID-19, a higher 30-day mortality was found compared with patients without COVID-19 infection. Perry et al. showed that COVID-19 at the onset of stroke was independently associated with death during admission (32). The preliminary analysis of a North American neurovascular consortium comprising 14 comprehensive stroke centers in the USA and Canada suggests that the rates of mortality with COVID-19-associated stroke are greater than in COVID-19 or stroke in otherwise. Mortality was significantly higher in African-Americans compared with other races (33). In the Italian population, patients who died in March–May 2020, compared with those who died in June–August 2020, had significantly lower rates of multiple comorbidities (three or more comorbidities) (34). We did not find such relationship. The meta-analysis of 16 studies involving 4,448 patients with cerebrovascular and cardiovascular diseases by Pranata et al. found that the presence of COVID-19 infection in patients with cerebrovascular disease was associated with an unfavorable outcome, while in patients with cardiovascular diseases with an unfavorable outcome, a higher mortality and a more severe course of COVID-19 infection were observed (35).

A previous publication reported that the restrictions taken to control the rapid spread of COVID-19 resulted in a sudden, unprecedented change in the lifestyle of individuals, leading to negative consequences on general health (36). We recognized this problem in the decline of patients reporting regular physician care (37).

COVID-19 infections among medical staff significantly impaired the care of patients with stroke. In our stroke team, almost half of the staff were infected. This was mainly due to a lack of experience of the staff in terms of care for patients with serious infectious disease and to the lack of sufficient personal protective equipment. Other publications highlighted the significant problem of COVID-19 infection among staff (38–40).

The current study has several limitations. Firstly, it is a retrospective single-center study, although data were collected prospectively. Therefore, the study group was limited in its number. Secondly, the small number of diagnosed and confirmed COVID-19 cases during the first wave of the pandemic was also related to a lack of experience of the medical personnel in diagnosing the disease as well as the unavailability and imperfection of diagnostic tests. Thirdly, in patients with stroke in the course of COVID-19 infection, we had only the possibility of 24-h Holter ECG monitoring, which is not in line with current recommendations, but this is only what can be refounded by the Polish health system. The abbreviated monitoring procedure could reduce the patient population with detected atrial fibrillation (41). Another limitation of the study was the lack of radiological assessment of the site lesion in ischemic stroke. We could not assess the impact of vaccinations on the occurrence and course of cerebrovascular incidents during the second wave of the pandemic, as vaccination in Poland began at the end of December. This problem should be assessed in a future study.

To conclude, cerebrovascular patients infected with COVID-19 were characterized by a higher rate of diagnosis of LVO and 30-day mortality. Despite the greater number of COVID-19 infections during the second wave of the pandemic, the work of the stroke center was better organized, which resulted in the higher percentage of reperfusion procedures, regardless of the frequent infections among SU staff. The greater number of patients treated during the second wave than during the first wave of the pandemic resulted from the lack of interruptions in the operation of our stroke center, creating buffer wards dedicated to infectious patients in our hospital, better organization of transport between hospitals, and reduction of fear of the medical staff of severe COVID-19 infection. However, dedicated staff training and better personal protective equipment are needed to prevent COVID-19 infection among the stroke unit staff.

## Data Availability Statement

The original contributions presented in the study are included in the article/supplementary material, further inquiries can be directed to the corresponding author.

## Ethics Statement

The studies involving human participants were reviewed and approved by Ethics Commttee of Collegium Medicum Jan Kochanowski University in Kielce. The patients/participants provided their written informed consent to participate in this study.

## Author Contributions

PS designed and directed the study. PS, WS, DG-W, JW, KP, MA, TF-P, MK, and GK analyzed data. PS, WS, DG-W, JW, KP, MA, TF-P, and MK recruited the participants and acquired the data. PS, WS, DG-W, JW, and GK drafted the manuscript together. All authors revised and approved the final manuscript.

## Conflict of Interest

The authors declare that the research was conducted in the absence of any commercial or financial relationships that could be construed as a potential conflict of interest.

## Publisher's Note

All claims expressed in this article are solely those of the authors and do not necessarily represent those of their affiliated organizations, or those of the publisher, the editors and the reviewers. Any product that may be evaluated in this article, or claim that may be made by its manufacturer, is not guaranteed or endorsed by the publisher.
